# Clinical Factors Associated with Longer Hospital Stay Following Ovarian Cancer Surgery

**DOI:** 10.3390/healthcare7030085

**Published:** 2019-07-03

**Authors:** Christopher G. Smith, Daniel L. Davenport, Justin Gorski, Anthony McDowell, Brian T. Burgess, Tricia I. Fredericks, Lauren A. Baldwin, Rachel W. Miller, Christopher P. DeSimone, Charles S. Dietrich, Holly H. Gallion, Edward J. Pavlik, John R. van Nagell, Frederick R. Ueland

**Affiliations:** 1 Department of Obstetrics & Gynecology, University of Kentucky, Lexington, KY 40536-0293, USA; 2 Department of Surgery, University of Kentucky, Lexington, KY 40536-0293, USA; 3 Division of Gynecologic Oncology, Markey Cancer Center, University of Kentucky, Lexington, KY 40536-0293, USA

**Keywords:** ovarian cancer, length of stay, ACS-NSQIP, blood transfusion, neoadjuvant chemotherapy, interval debulking surgery, primary cytoreductive surgery

## Abstract

*Background*: Ovarian cancer (OC) is the leading cause of death from gynecologic malignancy and is treated with a combination of cytoreductive surgery and platinum-based chemotherapy. Extended length of stay (LOS) after surgery can affect patient morbidity, overall costs, and hospital resource utilization. The primary objective of this study was to identify factors contributing to prolonged LOS for women undergoing surgery for ovarian cancer. *Methods*: The American College of Surgeons National Surgical Quality Improvement Program (ACS-NSQIP) database was queried to identify women from 2012–2016 who underwent hysterectomy for ovarian, fallopian tube and peritoneal cancer. The primary outcome was LOS >50th percentile. Preoperative and intraoperative variables were examined to determine which were associated with prolonged LOS. *Results*: From 2012–2016, 1771 women underwent elective abdominal surgery for OC and were entered in the ACS-NSQIP database. The mean and median LOS was 4.6 and 4.0 days (IQR 0–38), respectively. On multivariate analysis, factors associated with prolonged LOS included: American Society of Anesthesiologists (ASA) Classification III (aOR 1.71, 95% CI 1.38–2.13) or IV (aOR 1.88, 95% CI 1.44–2.46), presence of ascites (aOR 1.88, 95% CI 1.44–2.46), older age (aOR 1.23, 95% CI 1.13–1.35), platelet count >400,000/mm^3^ (aOR 1.74, 95% CI 1.29–2.35), preoperative blood transfusion (aOR 11.00, 95% CI 1.28–94.77), disseminated cancer (aOR 1.28, 95% CI 1.03–1.60), increased length of operation (121–180 min, aOR 1.47, 95% CI 1.13-1.91; >180 min, aOR 2.78, 95% CI 2.13–3.64), and postoperative blood transfusion within 72 h of incision (aOR 2.04, 95% CI 1.59–2.62) (*p* < 0.05 for all). *Conclusions*: Longer length of hospital stay following surgery for OC is associated with many patient, disease, and treatment-related factors. The extent of surgery, as evidenced by perioperative blood transfusion and length of surgical procedure, is a factor that can potentially be modified to shorten LOS, improve patient outcomes, and reduce hospital costs.

## 1. Introduction

Ovarian cancer (OC) is the leading cause of gynecological cancer death for women in the United States [1]. In 2018, there were roughly 22,000 new cases of ovarian cancer and 14,000 deaths [2]. Over sixty percent of women diagnosed with ovarian cancer have either Stage III or IV disease [3] and are treated with a combination of cytoreductive surgery and platinum-based chemotherapy [4]. Surgery for advanced stage cancer can be extensive and often results in longer hospitalizations [5].

Length of hospital stay (LOS) following surgical cancer care is an important measure of short-term quality of care. Prolonged LOS increases medical resource utilization, overall cost, readmissions, and short-term mortality [6,7]. Longer hospitalizations are also associated with iatrogenic and hospital-acquired sequelae that contribute to increased morbidity and mortality [8]. Preoperative hypoalbuminemia (albumin <3.5 g/dL), body weight loss (>10 percent decrease in body weight six months prior to surgery), body mass index <18.5 kg/m^2^, dialysis status, disseminated cancer, increasing frailty, non-Caucasian race, perioperative anemia, blood transfusion, increasing operative time, age, and American Society of Anesthesiologists (ASA) classification have all been associated with prolonged LOS for various abdominal and gynecological surgeries [9,10,11,12,13,14,15,16,17].

The current literature is lacking regarding the perioperative factors affecting LOS for women undergoing surgery for ovarian cancer. The primary objective of this population-based quality assessment analysis is to understand the clinical factors associated with longer LOS following ovarian cancer surgery.

## 2. Materials and Methods

The American College of Surgeons National Surgical Quality Improvement Program (ACS-NSQIP) database was examined to identify patients who underwent surgery for ovarian, fallopian tube, and peritoneal cancer from 2012–2016.

The ACS-NSQIP database is a reporting system intended to provide reliable, risk-adjusted surgical outcomes data to surgical services and medical center administrators allowing the assessment and improvement of surgical quality at a national level. As previously reported, the ACS-NSQIP data are prospectively collected in a standardized fashion according to strict definitions by dedicated clinical data coordinators. Risk and outcome variables are meticulously defined, and coordinators complete in-depth training on all study definitions. Regular conference calls, annual meetings, and site visits are used to maintain data reliability. Patients are followed throughout their hospital stay and after hospital discharge up to 30 days postoperatively. Clinical data coordinators collect data from patients electronic and paper medical records, and patient telephone interviews. Additional operations within 30 days of an included case are excluded. Patients less than 18 years old and admissions for trauma are excluded. The accuracy and reproducibility of the data have been previously demonstrated [18,19,20,21].

Current Procedural Terminology (CPT^®^) codes were used to select patients from the ACS-NSQIP database who had undergone elective same-day surgery for ovarian, fallopian tube, and peritoneal cancers from 2012–2016 (Appendix A). International Classification of Diseases, 9th and 10th Revision (ICD-9, ICD-10) codes were used to identify patients with ovarian, fallopian tube, and peritoneal cancers, which will be collectively referred to in this manuscript as ovarian cancer (OC) (Appendix B).

Patient variables and complications were defined and abstracted according to the ACS-NSQIP methodology. Specifically, “disseminated malignancy” is defined by ACS-NSQIP as “patients who have cancer that: (1) Has spread to one site or more sites in addition to the primary site AND (2) in whom the presence of multiple metastases indicates the cancer is widespread, fulminant or near terminal.” “Ascites” is defined within the database as the presence of fluid accumulation in the peritoneal cavity noted on physical examination, abdominal ultrasound, or abdominal computed tomography (CT)/magnetic resonance imaging (MRI) within 30 days prior to surgery, or malignant ascites due to extensive cancer (excluding liver disease) [18].

For each patient, ACS-NSQIP collects LOS data which is defined as the length of hospitalization from the date of admission for elective same day surgery until discharge. Prolonged LOS was defined as LOS greater than the median.

Demographic, medical history, review of systems and laboratory values were compared between patients who had prolonged LOS and those who did not. Chi-square testing was performed for categorical variables, and Fisher’s exact tests were used for binary variables. Only variables with *p* < 0.05 for bivariate comparisons were included in the multivariable analysis. Stepwise logistic regression was used to determine independent risk factors for prolonged LOS. Significant risk factors identified in the bivariate analysis were considered for inclusion into the multivariate analysis if *p* < 0.05 and were removed from analysis if *p* > 0.10. Once independent preoperative risks were determined, intraoperative variables were identified in a similar step forward fashion while adjusting for the presence of preoperative variables [22]. The Hosmer–Lemeshow statistic reflects results of the Hosmer–Lemeshow test which tests the goodness of fit for logistic regression and is primarily used in risk prediction models; small values reflect a poorly fit model. The concordance (c) statistic was calculated to measure discrimination of the multivariable model. The c-statistic has values ranging from 0–1 and represent the area under the receiver operating characteristic curve. Values close to 0.5 indicate a poor model, 0.7–0.8 as good, while those values near 1 reflect a strong model.

## 3. Results

The ACS-NSQIP database contained 1771 patients who underwent same day elective surgery for OC from 2012–2016. Laparotomy was performed in all cases. Mean and median LOS was 4.6 and 4.0 days (IQR 0–38), respectively. For this study, prolonged length of stay was defined as >4 days.

The results of a bivariate analysis are listed in Table 1. Several non-modifiable risk factors were associated with prolong LOS, including older age (63.5 vs. 60.2, *p* < 0.001), American Society of Anesthesiologists (ASA) Classification III or IV (67.6% vs. 50.3%, *p* < 0.001), elevated hepatic transaminases (5.9% vs. 3.5%, *p* = 0.006), renal dysfunction (5.4% vs. 2.5%, *p* = 0.003), ascites (24.2% vs. 12.5%, *p* < 0.001), disseminated cancer (37.3% vs. 26.6%, *p* < 0.001), and recent weight loss (5.9% vs. 3.7%, *p* = 0.04). Other preoperative factors associated with longer LOS include blood transfusion (1.0% vs. 0.1%, *p* = 0.008), hematocrit <38% (58.8% vs. 53.2%, *p* < 0.03) and thrombocytosis (17.0% vs. 10.5%, *p* < 0.001).

Intraoperative variables associated with prolonged postoperative LOS include blood transfusion within 72 h of incision (33.8% vs. 15.0%, *p* < 0.001) and length of surgery (176 min vs. 146 min, *p* < 0.001), particularly operative times exceeding 180 min (Table 1).

The findings from the multivariate analysis are summarized in Table 2. Preoperative clinical factors associated with prolonged LOS include ASA Class III (aOR 1.71, 95% CI 1.38–2.13), ASA Class IV (aOR 1.88, 95% CI 1.44–2.46), ascites (aOR 1.88, 95% CI 1.44–2.46), older age (aOR 1.23, 95% CI 1.13–1.35), thrombocytosis (aOR 1.74, 95% CI 1.29–2.35), blood transfusion (aOR 11.00, 95% CI 1.28–94.77), and disseminated cancer (aOR 1.28, 95% CI 1.03–1.60). Other clinical factors associated with prolonged LOS include blood transfusion within 72 h of incision (aOR 2.04, 95% CI 1.59–2.62) and operative time >180 min (aOR 2.78, 95% CI 2.13–3.64). The multivariable model showed goodness of fit (Hosmer–Lemeshow statistic *p* = 0.398), as well as good discrimination of patients with prolonged LOS after surgery for ovarian cancer (c-statistic = 0.71, 95% CI = 0.69–0.74).

## 4. Discussion

Our investigation has identified an association between longer LOS and several clinical factors following surgery for ovarian cancer, some of which can be potentially impacted by medical or surgical decisions. OC is a disease of older women who frequently have numerous medical comorbidities like hypertension, diabetes mellitus, cardiovascular disease, and pulmonary disease [23,24]. Women generally present with advanced and disseminated cancer which is commonly associated with weight loss, severe malnutrition, and impaired gastrointestinal function, which can contribute to a protracted surgical recovery, and prolonged hospitalization [25]. Women with advanced stage disease typically undergo extensive cytoreductive surgery resulting in longer hospitalizations [3].

The extent of ovarian cancer surgery correlates with length of surgery, blood loss, and transfusion rate which we demonstrated to predict prolonged LOS (Table 2) [15,26,27]. Neoadjuvant chemotherapy (NACT), comprising of three (sometimes 4–6) cycles of platinum-based chemotherapy followed by interval debulking surgery (IDS) [5], is a compelling treatment approach that can impact some these controllable risk factors. Two recent randomized, controlled trials found equivalent overall survival and progression-free survival (30 vs. 29 months; 24.1 vs. 22.6 months (overall survival), 12 months; 10.7 vs. 12.0 months (progression-free survival)) with reduced surgical morbidity following NACT with interval debulking surgery (IDS) compared to PCS with the goal of both surgeries to resect all visible disease [28,29]. A recent meta-analysis comparing NACT to primary cytoreductive surgery (PCS) reported that NACT had a higher rate of complete cytoreduction (RR 1.95 (95% CI 1.33–2.87)), less perioperative infection (RR 0.30 (95% CI 0.16–0.56)), fewer gastrointestinal fistulae (RR 0.24 (95% CI 0.06–0.95)), fewer grade 3 or 4 adverse events (RR 0.29 (95% CI 0.11–0.78)), fewer postsurgical deaths (RR 0.14 (95% CI 0.04–0.49)), and better quality of life [30]. Furthermore, NACT has been shown to have decreased intraoperative blood losses, is less likely to receive perioperative blood transfusions with decreased number of units received, has earlier times to ambulation and return of intestinal function, and has shorter LOS [28,29,31,32,33,34]. NACT is also associated with significantly fewer intestinal diversions (10.8% vs. 23.3%, *p* < 0.0001) [35]. NACT is becoming increasingly common in the United States as quality and safety data continues to favor NACT over PCS [36]. Additionally, NACT is utilized more for women with high perioperative risk or unresectable disease, common characteristics of patients with OC [4,37].

Admission to an intensive care unit (ICU) is needed for 25–36% of women undergoing aggressive PCS for ovarian cancer [38]. Factors associated with ICU admission following surgery for a gynecologic cancer include many of the factors identified in this investigation relating to LOS, including advanced age, medical comorbidities, malnutrition, long operative times, blood loss and blood transfusions, as well as increased intraoperative fluid resuscitation and bowel resection with anastomosis [39,40]. PCS results in a nearly three-fold increase in ICU admissions and longer LOS when compared to NACT. Even though patients receiving NACT are generally older and have more advanced disease, ICU admission rates are significantly lower compared to PCS. Patient survival is also worse following unplanned ICU admissions, including a shorter median survival (27.3 vs. 57.9 months, *p* < 0.001, adjusted HR 2.16, 95% CI 1.53–3.05). The increased utilization of NACT will likely reduce the length and number of ICU admissions for women undergoing ovarian cancer surgery, while also decreasing overall LOS [5].

Patient morbidity and mortality are not the only considerations related to prolonged LOS following surgery for OC. It is well known that prolonged LOS contributes to increased cost for both patient and hospital [40,41]. Pragmatically, when LOS is shorter than the national average, the Medicare payment to the hospital is likely to exceed the actual cost of care. Thus, patients and hospitals both stand to benefit from shorter LOS [42,43].

This investigation has several strengths. The database identified a large cohort of women undergoing surgery for ovarian cancer. The data is prospectively collected from a well-established national database, and it is generalizable since it was collected from numerous medical facilities. Unfortunately, information for NACT was not included in the study because of insufficient numbers in the NSQIP database. Also, cancer stage and histology are variables not included in the NSQIP database, therefore specific stage and histology information is not provided in this manuscript. Additionally, the volume of blood loss, immediate pre- and postoperative hemoglobin/hematocrit concentrations, as well as the thresholds utilized for blood transfusion are not available for analysis. Other factors that may influence perioperative outcomes such as operating surgeon experience, surgical volume, and institution type are not able to be controlled using the ACS-NSQIP database but does lend to the generalizability of the findings. Furthermore, the study period for which hysterectomy data was available was before the publication of ERAS Pathways for gynecologic oncology surgery. As with all retrospective studies, information and selection biases may affect comparisons.

## 5. Conclusions

Our findings identify several important variables that are associated with longer hospitalizations following surgery for ovarian cancer. Older, sicker patients with clinical findings of advanced cancer, who undergo complex surgery (demonstrated by increased likelihood of blood transfusion, and longer surgeries), have a greater association with longer hospitalizations (Table 2). Some of these clinical factors can potentially be influenced by treatment-related decisions, specifically the extent of surgery, the surgical procedure itself, and possibly NACT. In the future, the increased use of the ACS-NSQIP database by gynecologic oncologists should allow for meaningful comparisons between PCS and NACT.

## Figures and Tables

**Table 1:** Preoperative and intraoperative variables of patients undergoing surgery for ovarian cancer, stratified by length of stay. Median length of stay was 4 days.

Variable	LOS 0–4 Day(s) (*n* = 1159)	LOS >4 Days (*n* = 612)	All Patients (*n* = 1771)	*p*-Value *
**Preoperative**				
Age, years (mean, standard deviation)	60.2 (11.9)	63.5 (11.8)	61.3 (12.0)	<0.001
Body Mass Index >30 kg/m^2^	404 (34.9)	235 (38.4)	639 (36.1)	0.145
ASA Classification				<0.001
1–2	577 (49.8)	198 (32.4)	775 (43.8)	
3	550 (47.5)	377 (61.6)	927 (52.3)	
4	32 (2.8)	37 (6.0)	69 (3.9)	
White Race	822 (70.9)	450 (73.5)	1272 (71.8)	0.012
Black	57 (4.9)	47 (7.7)	104 (5.9)	
Other	77 (6.6)	34 (5.6)	111 (6.3)	
Undocumented	203 (17.5)	81 (13.2)	284 (16.0)	
Hispanic Ethnicity	62 (5.3)	28 (4.6)	90 (5.1)	0.003
Unknown	195 (16.8)	68 (11.1)	263 (14.9)	
Not Hispanic	902 (77.8)	516 (84.3)	1418 (80.1)	
Diabetes, Non-insulin	100 (8.6)	65 (10.6)	165 (9.3)	0.059
Insulin	38 (3.3)	31 (5.1)	69 (3.9)	
Dyspnea	72 (6.2)	58 (9.5)	130 (7.3)	0.016
Ascites	145 (12.5)	148 (24.2)	293 (16.5)	<0.001
Disseminated Cancer	308 (26.6)	228 (37.3)	536 (30.3)	<0.001
Recent weight loss >10% body mass	43 (3.7)	36 (5.9)	79 (4.5)	0.040
Treated hypertension	461 (39.8)	304 (49.7)	765 (43.2)	<0.001
Preoperative blood transfusion	1 (0.1)	6 (1.0)	7 (0.4)	0.008
Preoperative SIRS/Septic Shock	8 (0.7)	9 (1.5)	17 (1.0)	0.126
Alkaline Phosphatase >125 IU/L	55 (4.7)	50 (8.2)	105 (5.9)	0.006
SGOT >40 U/L	41 (3.5)	36 (5.9)	77 (4.3)	0.027
Creatinine >1.2 mg/dL	29 (2.5)	33 (5.4)	62 (3.5)	0.003
Hematocrit <38%	617 (53.2)	360 (58.8)	977 (55.2)	0.027
Platelets >400,000/mL	122 (10.5)	104 (17.0)	226 (12.8)	<0.001
PTT >35 s	45 (3.9)	40 (6.5)	85 (4.8)	0.019
**Intraoperative**				
Wound Classification				0.842
1-Clean	69 (6.0)	36 (5.9)	105 (5.9)	
2-Clean/Contaminated	1042 (89.9)	547 (89.4)	1589 (89.7)	
3-Contaminated	48 (4.1)	29 (4.7)	77 (4.3)	
Duration of Operation, min. (mean, standard deviation)	146 (67)	176 (79)	156 (73)	<0.001
Duration of Operation, min.				
≤120	463 (39.9)	155 (25.3)	618 (34.9)	
121–180	403 (34.8)	200 (32.7)	603 (34.0)	<0.001
>180	293 (25.3)	257 (42.0)	550 (31.1)	
Transfusion within 72 h’s. of incision	174 (15.0)	207 (33.8)	381 (21.5)	<0.001

* Fisher exact test or chi-square test. *p* < 0.05 was considered statistically significant; ASA—American Society of Anesthesiologists; SIRS—systemic inflammatory response syndrome; SGOT—Aspartate transaminase; IU—international units; PTT—partial thromboplastin time.

**Table 2:** Multivariable predictors of prolonged LOS in patients undergoing hysterectomy for ovarian cancer (Hosmer-Lemeshow (H-L) statistic *p* = 0.398, c-index = 0.71).

Variable	Prevalence (%)	Adjusted Odds Ratio (95% CI)	*p*-Value *
Age per 10 years from mean	63.5 (11.8)	1.23 (1.13–1.35)	<0.001
ASA Classification III vs. I–II (ref.)	377 (61.6)	1.71 (1.38–2.13)	<0.001
IV	37 (6.0)	2.80 (1.68–4.66)	<0.001
Ascites	148 (24.2)	1.88 (1.44–2.46)	<0.001
Platelets >400,000/mL	104 (17.0)	1.74 (1.29–2.35)	<0.001
Preoperative blood transfusion	6 (1.0)	11.00 (1.28–94.77)	0.029
Disseminated cancer	228 (37.3)	1.28 (1.03–1.60)	0.028
Duration of Operation (mins)	200 (32.7)	1.47 (1.13–1.91)	0.004
≤120 vs. 121–180 (ref)			
>180	257 (42.0)	2.78 (2.13–3.64)	<0.001
Transfusion within 72 hrs. of incision	207 (33.8)	2.04 (1.59–2.62)	<0.001

* Fisher exact test or chi-square test. *p* < 0.05 was considered statistically significant; ASA—American Society of Anesthesiologists.

## Appendix A

**Table A1:** Primary current procedure terminology codes included in the study.

Primary CPT ^®^ Codes of Procedures Included in Study	
58951	Total abdominal hysterectomy/bilateral salpingo-oophorectomy, debulking, lymph node dissection
58953	Total abdominal hysterectomy/bilateral salpingo-oophorectomy, radical debulking
58954	Bilateral salpingo-oophorectomy with omentectomy, total abdominal hysterectomy and radical dissection for debulking; with pelvic lymphadenectomy and limited para-aortic lymphadenectomy
58956	Total abdominal hysterectomy/bilateral salpingo-oophorectomy, omentectomy
58957	Total abdominal hysterectomy/bilateral salipingo-oophorectomy, +/− omentectomy
58150	Total abdominal hysterectomy (corpus and cervix), with or without removal of tube(s), with or without removal of ovary(s)

## Appendix B

**Table A2:** International statistical classification of diseases and related health problems codes included in the study.

ICD-9 Codes Included in Study	
158.8	Malignant neoplasm of specified parts of peritoneum
158.9	Malignant neoplasm of peritoneum, unspecified
183.0	Malignant neoplasm of ovary
183.2	Malignant neoplasm of fallopian tube
183.3	Malignant neoplasm of broad ligament of uterus
183.4	Malignant neoplasm of parametrium
183.8	Malignant neoplasm of other specified sites of uterine adnexa
183.9	Malignant neoplasm of uterine adnexa, unspecified site
**ICD-10 Codes included in study**	
C48.2	Malignant neoplasm of peritoneum, unspecified
C56	Malignant neoplasm of ovary
C57	Malignant neoplasm of other and unspecified female genital organs
C57.4	Malignant neoplasm of uterine adnexa, unspecified
D39.1	Neoplasm of uncertain behavior of ovary
D39.11	Neoplasm of uncertain behavior of right ovary
D39.12	Neoplasm of uncertain behavior of left ovary
